# Lysis profile and preference of *Myxococcus* sp. PT13 for typical soil bacteria

**DOI:** 10.3389/fmicb.2023.1211756

**Published:** 2023-06-12

**Authors:** Yi Yang, Hong Tao, Wenwen Ma, Nana Wang, Xiaolin Chen, Wenhui Wang

**Affiliations:** School of Life Sciences, Anhui Agricultural University, Hefei, Anhui, China

**Keywords:** myxobacteria, prey bacteria, predation diameter, microcosmic system, bacterial community structure

## Abstract

**Introduction:**

*Myxococcus* sp. PT13 is a wild strain with multiple predatory properties that prey on multiple model microorganisms preserved in the laboratory. However, the lysis spectrum of PT13 on typical soil bacteria and its driving effect on soil microecosystems are still unclear.

**Methods:**

In this study, the lawn predation method was used to determine the predation diameter of 62 typical soil bacteria by myxobacteria PT13 and analyze their lysis spectra.

**Results and Discussion:**

The results showed that PT13 had a predation diameter greater than 15 mm against typical soil microorganisms such as *Aeromonas*, *Bacillus, Brevibacterium*, *Fictibacillus*, *Glutamicibacter*, *Herbaspirillum,* and *Leifsonia* and had an outstanding lysis effect but a significant preference (*p* < 0.05). Absolute high-throughput sequencing results showed that PT13 predation drove the microcosmic system composed of 16 bacterial genera, with a significant decrease in the Shannon index by 11.8% (CK = 2.04, *D* = 1.80) and a significant increase in the Simpson index by 45.0% (CK = 0.20, *D* = 0.29). The results of principal coordinate analysis (PCoA) showed that myxobacterial addition significantly disturbed the microcosmic microbial community structure (ANOSIM, *p* < 0.05). LEfSe analysis showed that the relative and absolute abundances (copy numbers) of *Bacillus*, *Pedobacter*, *Staphylococcus*, *Streptomyces* and *Fictibacillus* decreased significantly very likely due to myxobacterial predation (*p* < 0.05). However, the predatory effect of PT13 also increased the relative or absolute abundances of some species, such as *Sphingobacterium*, *Paenarthrobacter*, *Microbacterium,* and *Leifsonia*. It can be concluded that PT13 has a broad-spectrum lysis spectrum but poor cleavage ability for *Streptomyces*, and the interaction between complex microorganisms limits the predation effect of PT13 on some prey bacteria. This in turn allows some prey to coexist with myxobacteria. This paper will lay a theoretical foundation for the regulation of soil microecology dominated by myxobacteria.

## Introduction

Myxobacteria are typical indigenous predatory bacteria that are species-rich, globally distributed and inhabit a wide range of natural environments, such as soils. They prefer to inhabit non-saline soils and sediments, some prefer saline environments and rarely occur in host-associated environments (Wang et al., 2021). Myxobacteria are difficult to isolate and purify due to their intrinsic characteristics, and currently, prey-baiting isolation is an important method to obtain myxobacteria (Yi et al., 2021). Mostly gram-negative bacteria, but also some gram-positive bacteria, can induce myxobacteria fruiting bodies well to achieve myxobacteria strain isolation (Zhou et al., 2020). Among the known bacterial groups, myxobacteria has the largest known genome (some reaching 13–14 Mb), packed with numerous specialized metabolite biosynthetic gene clusters (BGCs) (Phillips et al., 2022). They are considered to be a rich source of secondary metabolites, mainly antibiotics and lytic enzymes with bacterial lysis and cellulolytic properties. Based on this feature, myxobacteria often have great potential for the production of novel drugs (Bhat et al., 2021).

Myxobacteria can feed on living microbial cells or other biomolecules to obtain nutrients. Most strains form fruiting bodies through directed cell movement after nutrient depletion. The fruiting body contains a large number of stress-resistant myxospores, allowing them to survive in harsh environments (Liu et al., 2019). Thus, myxobacteria have a complex life history and growth metabolism regulation process and good environmental adaptability (Li et al., 2021). They mostly adopt collaborative predation, also known as the “wolf pack attack” strategy, in which they lyse prey with antibiotics and hydrolytic enzymes (Pérez et al., 2016). The predation process may be regulated by the motor system, chemotaxis system, secretion of proteases and antimicrobial substances, and intercellular signaling system (Berleman and Kirby, 2009). They can find prey by recognizing *in vitro* acyl homoserine lactones (AHLs) secreted by prey and hunt efficiently through adventure and social movements (Lloyd and Whitworth, 2017; Whitworth and Zwarycz, 2020; Shukria et al., 2021).

Predation is a key process in building ecosystem communities and maintaining biodiversity, and predators can exert an important influence on ecosystems. Studies have shown that myxobacteria are common soil predators and may even be dominant (Petters et al., 2021). Their body size is more similar to the size of prey bacterial cells, making it more convenient to prey on bacteria (Petters et al., 2021). Myxobacteria prey on other soil bacteria and fungi, driving the proportion of bacteria in the soil. They are also new biocontrol microorganisms that prevent and control plant pathogenic fungi and bacteria (Bull et al., 2002; Ye et al., 2020; Li et al., 2022). A study in which the authors of this paper participated showed that the predation ability of *Corallococcus* sp. strain EGB on 9 different prey bacteria was significantly different. The volume of EGB added in simple artificial microcosmic systems is a major factor in the change in microbial community structure (Dai et al., 2020).

Soil is the base camp of microorganisms, and myxobacteria are considered to be indigenous bacteria with broad-spectrum predation ability in soil. *Myxococcus* sp. PT13 is a wild strain isolated from yellow-brown soil collected from Huangshan City, Anhui Province, China, using *Escherichia coli* as bait (Yi et al., 2021), and the soil samples used for PT13 isolation were consistent with the soil samples in the material method. It can prey on many strains of bacteria and fungi preserved in the laboratory, but the preference of PT13 to prey on indigenous prey bacteria and its driving effect on soil microecosystems are unclear. Therefore, this study mainly aimed to (1) clarify the predation preference of PT13 for indigenous bacteria and explore its potential for restoring soil biodiversity and ecological functions; (2) build a research system based on predation by myxobacteria to provide a model reference for further ecological function research; and (3) enrich the knowledge and understanding of predatory myxobacteria in soil systems and provide theoretical and technical support for their application in agriculture and medicine.

## Materials and methods

### Soil sampling and isolation of prey bacteria

The soil samples used for this experiment were collected from yellow–brown soil in Huangshan City, Anhui Province, China (30°23′N, 118°12′E), which belongs to the subtropical monsoon climate with an average annual temperature of 15–16°C, an average annual rainfall of 1,670 mm and a frost-free period of 236 days. Five kilograms of soil was collected at a depth of 20 cm and sieved (1 cm × 1 cm) to remove plant and other debris. The collected soil samples were stored in a refrigerator at 4°C. Then, 10 g of fresh soil sample was weighed and shaken in a sterile conical flask containing 90 mL of sterile water and glass beads at 180 rpm on a shaker for 2 h. The gradient dilution plate method (Liu et al., 2021) was used to coat the soil suspension to LB (Tryptone 1 g, Yeast extract 0.5 g, NaCl 1 g, Agar 2 g, H_2_O 100 ml) and Gao’s No.1 (Soluble Starch 2 g, KNO_3_ 0.1 g, K_2_HPO_4_•3H_2_O 0.05 g, MgSO_4_•7H_2_O 0.05 g, NaCl 0.05 g, FeSO_4_•7H_2_O 0.001 g, Agar 2 g, H_2_O 100 ml) plates, and plates were incubated at 37, 30, or 25°C.

Single colonies of different colors and morphologies were selected on solid medium where the bacterial colonies were grown and inoculated into the same medium using plate streaking. DNA from the above bacterial colonies was extracted and the 16S rRNA gene was amplified by PCR. Amplification product sequencing was performed by Tsingke Biotechnology Co., Ltd., and BLAST was performed on NCBI to obtain taxonomic information. The phylogenetic tree was constructed using MEGA 7.0^1^ from the amplification sequences and visualized by Interactive Tree of Life (iTOL, version 4.3.2) (Letunic and Bork, 2016).

### Predation experiments

PT13 was inoculated into CYE liquid medium (1 g casein peptone, 0.5 g yeast extract, 0.1 g MgSO_4_•7H_2_O, 100 ml H_2_O) and incubated at 30°C for 1 ~ 2 days. The prey bacterial strains were inoculated into LB or Gao’s No. 1 medium and incubated at 37°C or 30°C for 1 ~ 2 days. Bacteria were harvested by centrifugation, and the cell pellet was washed twice with TPM [10 mM Tris–HCl (pH 7.6), 1 mM KH_2_PO_4_, 8 mM MgSO_4_, 1% agarose] medium. Prey bacterial cultures were resuspended in TPM medium to 1 × 10^9^ cells/ml, and the myxobacterial cells were concentrated to a final cell density of 1 × 10^10^ cells/ml. The lawn predation method was used to determine the predation ability of PT13 against various soil bacteria (Mendes-Soares and Velicer, 2013; Li et al., 2018; Arend et al., 2021). Myxobacterial and prey bacterial cultures were resuspended in TPM medium [10 mM Tris–HCl (pH 7.6), 1 mM KH_2_PO_4_, 8 mM MgSO_4_, 1% agarose], and 200 μl of prey bacteria was spotted onto TPM solid medium. When the prey bacteria were air-dried to form lawns, 2 μl of PT13 was inoculated in the center of the lawn. Three sets of biological replicates were set up per experiment and incubated at 30°C for 4 days.

### Construction of the prey bacterium microcosm system

Prey strains in the logarithmic growth stage were centrifuged at 7000 rpm for 5 min. The bacterial body was retained and washed with TPM liquid medium and resuspended. The concentration of each bacterial solution was determined by the dilution gradient coating plate method or real-time quantitative PCR, as shown in Table 1. Then, 100 mL of each prey bacterial solution resuspended by the above 16 strains was added to a sterile 3 L blue cap bottle and allowed to stand at 30°C for 24 h to construct a prey bacteria mixture. PT13 in the logarithmic growth phase were treated in the same way as the predator. These prey bacteria mixture and predator constructed a 100 ml microcosmic system. In a microcosmic system, 80 ml of the above prey bacteria mixture was added to a sterile Erlenmeyer flask. Then, 0 mL (CK), 1 mL (A), 5 mL (B), 10 mL (C), and 20 mL (D) PT13 were added to different groups of Erlenmeyer flask, respectively. Finally, the volume was brought up to 100 ml with TPM liquid medium; four parallel replicates were conducted per group.

**Table 1:** Related information of 16 strains of prey bacteria and the concentration of diluted bacterial solution.

Type of strain	Number	Taxonomy	OD_600_	CFU/ml
*Pseudomonas resinovorans*	TB	*Bacteria; Proteobacteria; Gammaproteobacteria; Pseudomonadales; Pseudomonadaceae; Pseudomonas*	O.626A	6.0 × 10^7^
*Comamonas sediminis*	TE	*Bacteria; Proteobacteria; Betaproteobacteria; Burkholderiales; Comamonadaceae; Comamonas*	0.592A	8.5 × 10^8^
*Brevundimonas diminuta*	TG	*Bacteria; Proteobacteria; Alphaproteobacteria; Caulobacterales; Caulobacteraceae; Brevundimonas*	0.647A	5.6 × 10^8^
*Sphingobacterium mizutaii*	TJ	*Bacteria; Bacteroidetes; Sphingobacteriia; Sphingobacteriales; Sphingobacteriaceae; Sphingobacterium*	0.588A	7.8 × 10^8^
*Bacillus aerius*	TM	*Bacteria; Firmicutes; Bacilli; Caryophanales; Bacillaceae; Bacillus*	0.699A	2.2 × 10^8^
*Stenotrophomonas bentonitica*	TQ	*Bacteria; Proteobacteria; Gammaproteobacteria; Lysobacterales; Lysobacteraceae; Stenotrophomonas*	0.646A	7.7 × 10^8^
*Mitsuaria chitosomitabida*	TU	*Bacteria; Proteobacteria; Betaproteobacteria; Burkholderiales; Comamonadaceae; Mitsuaria*	0.631A	6.72 × 10^9^
*Fictibacillus phosphorivorans*	TX	*Bacteria; Firmicutes; Bacilli; Bacillales; Bacillaceae; Fictibacillus*	0.678A	1.3 × 10^7^
*Staphylococcus aureus*	YC	*Bacteria; Firmicutes; Bacilli; Caryophanales; Staphylococcaceae; Staphylococcus*	0.669A	2.03 × 10^9^
*Pedobacter rhizosphaerae*	MA	*Bacteria; Bacteroidetes; Sphingobacteriia; Sphingobacteriales; Sphingobacteriaceae; Pedobacter*	0.617A	4.2 × 10^8^
*Delftia tsuruhatensis*	MK	*Bacteria; Proteobacteria; Betaproteobacteria; Burkholderiales; Comamonadaceae; Delftia*	0.695A	5.8 × 10^8^
*Brevibacterium sanguinis*	FD	*Bacteria; Actinobacteria; Actinobacteria; Micrococcales; Brevibacteriaceae; Brevibacterium*	0.670A	2.1 × 10^8^
*Paenarthrobacter nicotinovorans*	FI	*Bacteria; Actinobacteria; Actinomycetia; Micrococcales; Micrococcaceae; Paenarthrobacter*	0.510A	7.71 × 10^9^
*Leifsonia soli*	FN	*Bacteria; Actinobacteria; Actinomycetia; Micrococcales; Microbacteriaceae; Leifsonia*	0.694A	2.3 × 10^8^
*Glutamicibacter arilaitensis*	FP	*Bacteria; Actinobacteria; Actinomycetia; Micrococcales; Micrococcaceae; Glutamicibacter*	0.572A	1.0 × 10^7^
*Streptomyces cinnamonensis*	FS	*Bacteria; Actinobacteria; Actinomycetia; Streptomycetales; Streptomycetaceae; Streptomyces*	0.625A	8.08 × 10^7^#

^#^Representative qPCR result. CFU, colony-forming unit.

### Extraction and sequencing of sample DNA

After 12 h (T1) and 24 h (T2) of incubation, 1 ml of sample was added to a centrifuge tube and stored at −80°C for sequencing, and then 1 ml of sample was taken to determine its OD_600_ value. DNA was extracted using the FastDNA® SPIN Kit for soil (MP Biomedicals, Santa Ana, CA) according to the instructions and stored at −80°C. The V4-V5 region of the 16S rRNA gene (Primer F: GTGCCAGCMGCCGCGG, Primer R: CCGTCAATTCMTTTRAGTTT) was the target for absolute high-throughput sequencing by Shanghai Tianhao Biotechnology Co., Ltd. using the Illumina MiSeq PE250 (Wang et al., 2020b). These sequence data have been submitted to the GenBank database under accession number PRJNA953930.

### Statistical analyses

Quality control and bioinformatics analysis were performed using the DADA2 plug-in for QIIME2. Gene copy number was estimated for each amplicon sequence variant (ASV) based on the rrnDB database (version V5.6) (Stoddard et al., 2015). Species annotation of ASV sequences was performed using QIIME2 software (cutoff = 0.8). Principal coordinate analysis (PCoA) and analysis of similarities (ANOSIM) based on Bray–Curtis dissimilarities were performed using R (Version 4.1.0, vegan package) to assess the statistically significant effects of treatment processes on bacterial communities (Anderson and Walsh, 2013; Krych et al., 2013). Then, LEfSe was used to find specialized indicator bacterial groups within the different treatments of samples (Segata et al., 2011). All statistical analyses were performed by the R *stats* package (Version 4.1.0).

## Results

### Isolation of typical soil bacteria

**Figure 1:**
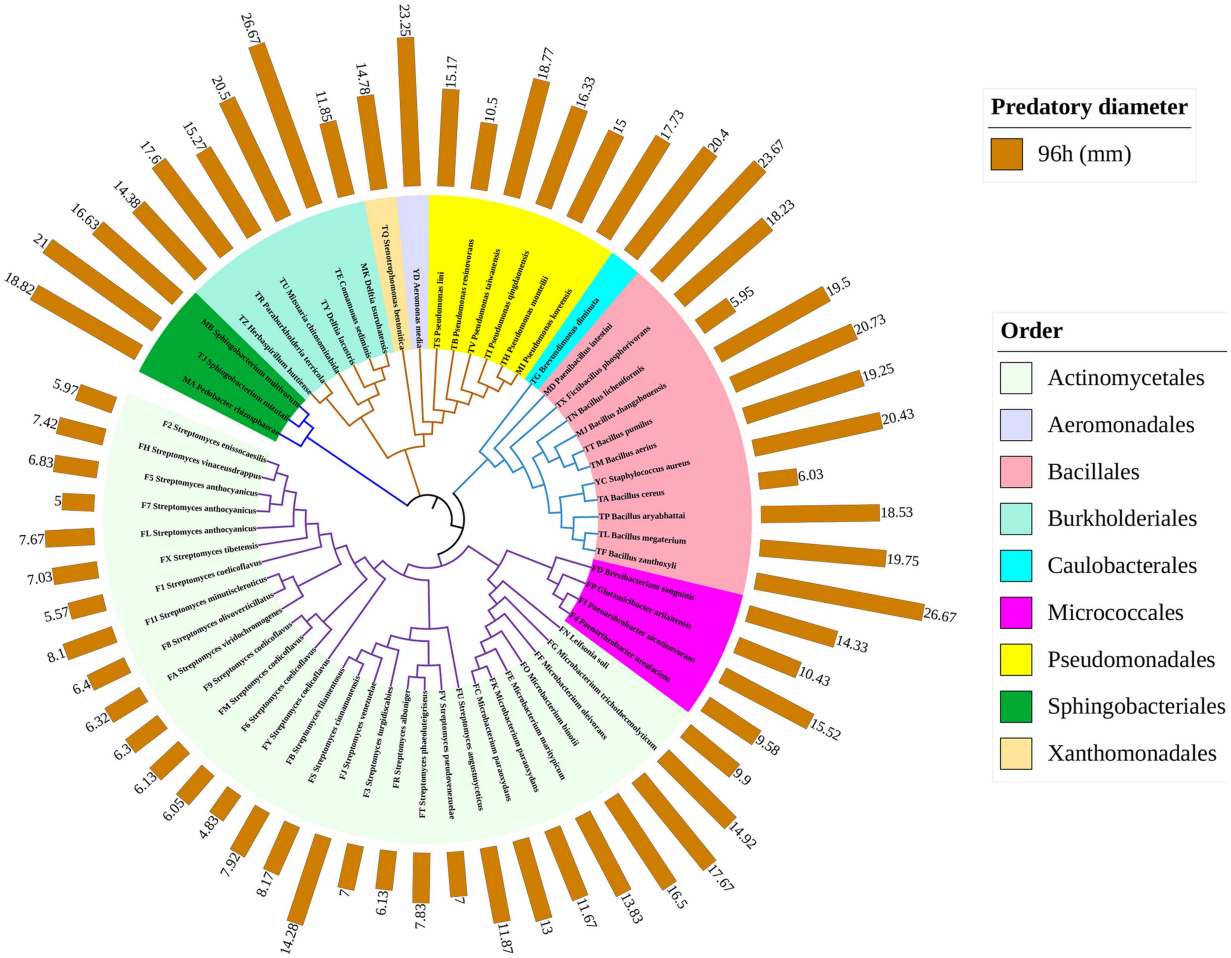
Phylogenetic tree based on the 16S rRNA gene sequences of 62 isolates. Phylogenetic tree branches are colored according to the bacterial order to which the species belongs. The outer orange bars represent the predation diameter (mm) of each strain at 96 h.

A total of 62 indigenous bacterial strains belonging to 21 different genera were isolated and purified from the soil samples (Supplementary Table S1). Among them were *Streptomyces* (22 strains), *Bacillus* (8), *Pseudomonas* (6), *Microbacterium* (6), *Paenarthrobacter* (2), *Sphingobacterium* (2)*, and Delftia* (2). One strain was isolated from each of the following genera: *Comamonas*, *Brevundimonas*, *Stenotrophomonas*, *Paraburkholderia*, *Mitsuaria*, *Pedobacter*, *Herbaspirillum*, *Paenibacillus*, *Fictibacillus*, *Staphylococcus*, *Aeromonas*, *Brevibacterium*, *Glutamicibacter*, and *Leifsonia*. A phylogenetic tree was constructed based on the 16S rRNA sequences of the above 62 strains. These 62 strains belong to the phyla Actinomycetes (33), Proteomycetes (15), Firmicutes (11) and Bacteroides (3), all of which are the dominant bacterial phyla in soil microorganisms (Figure 1).

### Predation experiments

**Figure 2:**
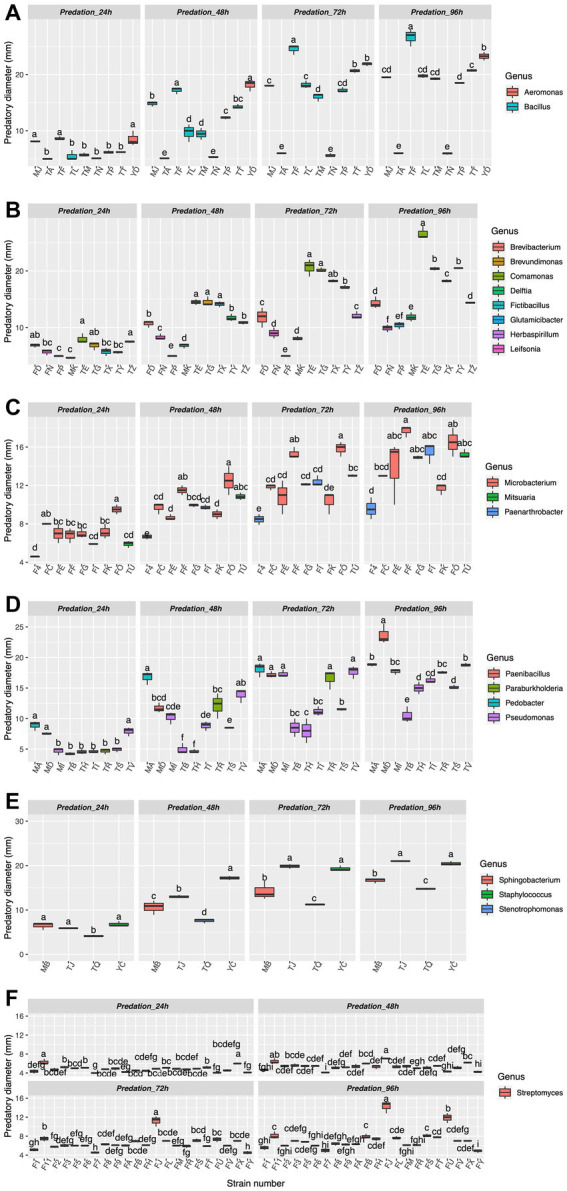
Myxobacteria PT13 predation on 62 strains of prey bacteria. The abscissa of the boxplot represents different strain number, and the ordinate represents the predation diameter at different culture times. Bacteria of different genera are distinguished by different colors, and **(A–F)** diagrams are made according to the alphabetical order of bacterial names. Different letters in the same diagram indicate a significant difference (ANOVA, *n* = 3, *p* < 0.05).

In TPM medium, which contains a lawn of prey bacteria as the only nutrient source, PT13 had a predatory effect on all 62 strains. The predation diameter of PT13 on prey bacteria increased with predation time. Some strains had prey diameters up to 20 mm at 96 h, such as *Aeromonas* and *Bacillus* (Figure 2A). However, there was a significant difference in predation diameter between the eight *Bacillus* strains in the same genus. The diameter of *Bacillus* sp. TF preyed upon by PT13 was 26.7 mm, which was significantly higher than that of the other seven *Bacillus* strains (*p* < 0.05). *Delftia* sp. TY and MK also had significantly different predation diameters of 20.5 and 11.9 mm, respectively (Figure 2B, *p <* 0.05). Significant differences in predation diameter between strains of the same genus were also observed in *Microbacterium*, *Paenarthrobacter,* and *Pseudomonas* (Figures 2C,D, *p* < 0.05).

Predation diameters varied more significantly between different bacterial genera. *Comamonas* sp. TE, *Brevundimonas* sp. TG and *Delftia* sp. TY all had predation diameters significantly larger than those of *Brevibacterium*, *Fictibacillus*, *Glutamicibacter*, *Herbaspirillum*, and *Leifsonia,* with a diameter at predation greater than 20 mm (Figure 1B, *p* < 0.05). Microorganisms with predation diameters greater than 15 mm mainly included *Paenibacillus*, *Paraburkholderia*, *Pedobacter*, *Sphingobacterium*, and *Staphylococcus* (Figures 2C–E).

In contrast, for 22 strains of *Streptomyces*, PT13 showed only a weak lytic capacity. The predation diameter of all *Streptomyces* spp. at 24, 48, and 72 h was less than 8 mm, except that the diameter of FJ was 11.2 mm at 72 h (Figures 1, 2). At 96 h, *Streptomyces* sp. FJ and FU had predation diameters of 14.3 mm and 11.9 mm, respectively, but those of the other 20 *Streptomyces* spp. were only 4–8 mm (Figures 1, 2). These results showed that PT13 had obvious predatory effects on the above indigenous bacteria, but its predation preference was directly related to the bacterial species.

### Microcosmic systems under predation by myxobacteria

**Figure 3:**
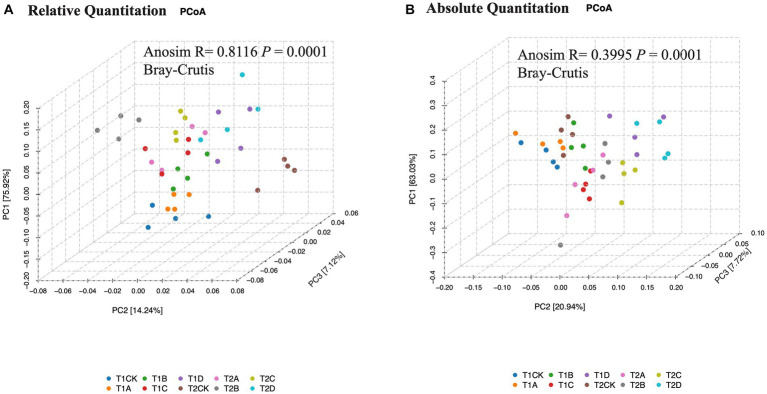
Effects of different volumes of myxobacteria treatment on the β diversity of bacterial community abundance **(A)** and copy number **(B)**. PCoA (Bray–Curtis distance index) plots allowing visualization of the differences in the bacterial community structure between samples (based on OTU information). The color of the sample points indicates the 10 treatments. The different sample numbers indicate the incubation time and the amount of PT13 added. T1: 12 h; T2: 24 h; CK: 0 ml of PT13; A: 1 ml; B: 5 ml; C: 10 ml; D: 20 ml.

In a microcosmic system with PT13 as its predator, different myxobacteria volumes significantly altered bacterial community structure α and β diversity (Supplementary Figures S1, S2 and Figure 3). Increased or decreased sequentially with the addition of PT13. At 12 h, the Shannon index of each group was 2.04, 2.04, 1.92, 1.84, and 1.80, decreasing sequentially with the addition of PT13 (ANOVA, *p* < 0.01). The Simpson index increased sequentially to 0.20, 0.21, 0.25, 0.28, and 0.29 (*p* < 0.01). The changes in the Shannon and Simpson indices at 24 h were similar to those at 12 h (*p* < 0.01). There were no significant differences between groups in the Chao1 and ACE indices (*p* < 0.01), indicating that predation of PT13 had no significant effect on species number at either incubation time.

PCoA demonstrated that 75.9 and 14.2% of the total community variation in relative abundance and 63.0 and 20.9% of the total community variation in absolute abundance was explained by PCoA1 and PCoA2, respectively. Figure 3A shows the difference between treatments with added myxobacteria (B, C and D) and CK in the direction of the PC1 axis, and Figure 3B shows the difference in the PC2 axis (Tables 2, 3, ANOSIM, Bray–Curtis, *p* < 0.01). This difference increased with the addition of PT13, indicating that the predation of myxobacteria PT13 was the main factor driving the composition of bacterial communities in the microcosmic system.

**Figure 4:**
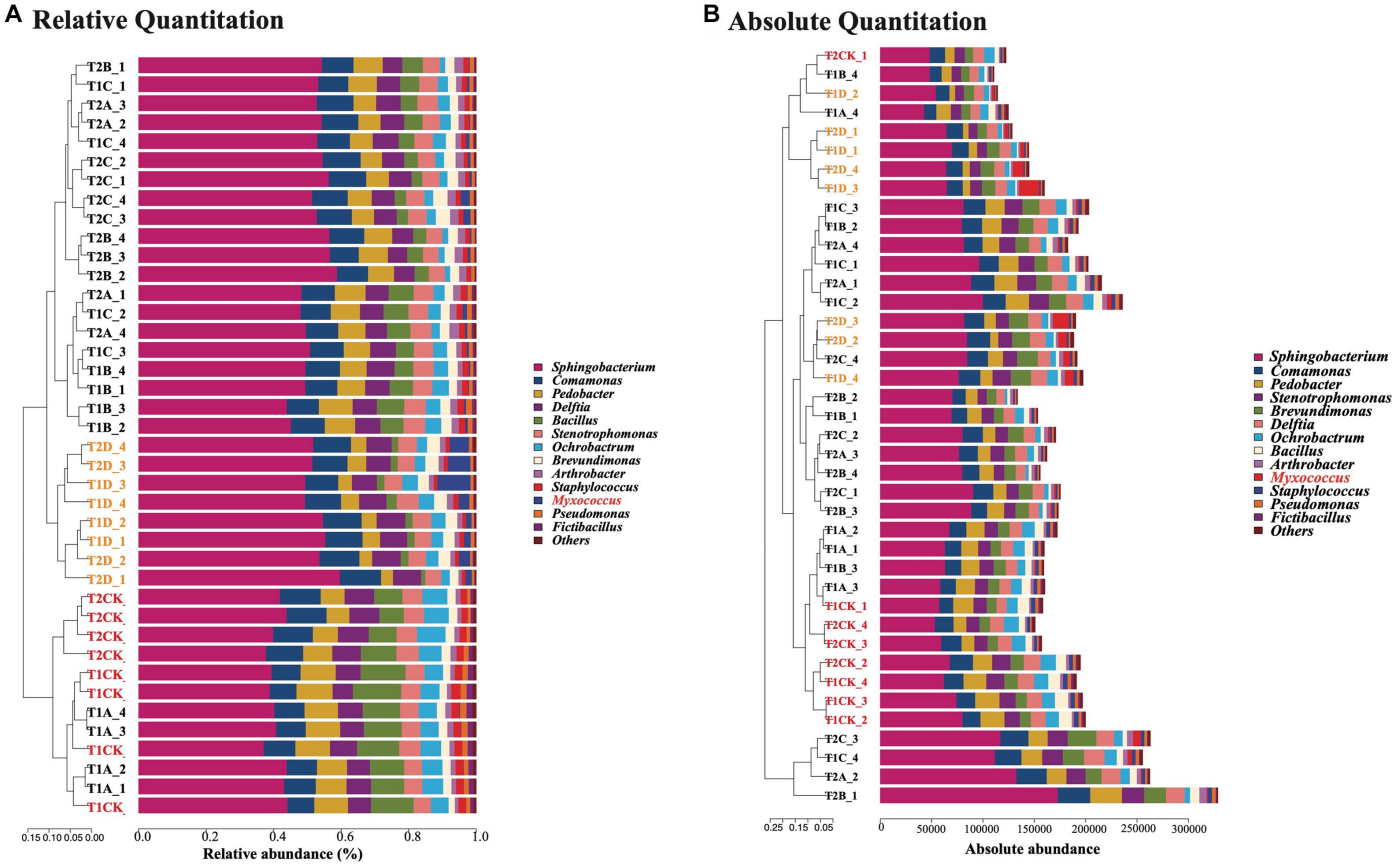
Relative **(A)** and absolute **(B)** abundance (copy number) of each genus under different volumes of myxobacteria. The different sample numbers indicate the incubation time and the volume of PT13 added. T1: 12 h; T2: 24 h; CK: 0 ml of PT13; A: 1 ml; B: 5 ml; C: 10 ml; D: 20 ml. The sample font of the control group (CK) is marked in red; the D group is labeled orange.

In the microcosmic system, *Sphingobacterium*, *Comamonas*, *Pedobacter*, *Delftia*, *Bacillus*, *Stenotrophomonas*, *Ochrobactrum*, *Brevundimonas*, *Paenarthrobacter*, and *Staphylococcus* were the 10 bacterial genera with the highest relative abundances. The relative abundances of *Myxococcus*, *Pseudomonas,* and *Fictibacillus* were lower. In the T1CK and T2CK treatments without myxobacteria, the abundance ratios of these genera did not change significantly between 12 and 24 h (Figure 4A). However, predation by PT13 drove community changes in the microcosm system, particularly at high volumes of D treatments. The abundance of *Sphingobacterium* increased significantly to 52.2 and 53.8% in the T1D and T2D groups, which increased by 30.8 and 31.5% compared with T1CK (39.9%) and T2CK (40.9%). The average abundance of PT13 in T1D and T2D was 4.2 and 4.4%, respectively. There was also a significant decrease in the abundance of *Pedobacter*, *Bacillus*, *Ochrobactrum,* and *Staphylococcus* in these groups.

The results of absolute abundance (copy number) were similar to the results of relative abundance. However, some samples did not show a significant decrease in total bacterial copy number with myxobacterial predation, such as T1C and T2C (Figure 4B). Under the predation of PT13, the absolute copy number of *Sphingobacterium* increased significantly, while that of *Pedobacter*, *Bacillus*, *Ochrobactrum,* and *Staphylococcus* decreased significantly. This indicated that the predation of PT13 caused the death of some bacteria but also nourished other types of bacteria, causing fluctuations in the total copy number of bacteria.

**Table 2:** ANOSIM analysis between five experimental treatments at 12 h.

	CK		A		B		C		D	
	*R*	*p*	*R*	*p*	*R*	*p*	*R*	*p*	*R*	*p*
CK			**0.438**	**0.091**	**0.979**	0.032*	**1.000**	0.028*	**1.000**	0.028*
A	0.240	0.091			**0.844**	0.029*	**1.000**	0.030*	**1.000**	0.030*
B	0.375	0.061	0.052	0.424			**0.26**	**0.145**	**0.854**	0.029*
C	0.833	0.027*	0.917	0.027*	0.490	0.026*			**0.729**	0.029*
D	0.781	0.031*	0.604	0.029*	0.010	0.483	0.708	0.030*		

Analysis of similarity was calculated between all treatments based on OTUs tables relative (bold font) and absolute abundance (regular font) Bray–Curtis distance matrices. Each pairwise comparison of two groups was performed using 999 permutations. *R* values > 0.75 are generally interpreted as clearly separated. *R* > 0.5 as separated and *R* < 0.25 as groups hardly separated (Krych et al., 2013). CK: 0 ml of PT13; A: 1 ml; B: 5 ml; C: 10 ml; D: 20 ml. *p < 0.05.

**Table 3:** ANOSIM analysis between five experimental treatments at 24 h.

	CK		A		B		C		D	
	*R*	*p*	*R*	*p*	*R*	*p*	*R*	*p*	*R*	*p*
CK			**1.000**	0.032*	**1.000**	0.028*	**1.000**	0.030*	**1.000**	0.027*
A	0.490	0.057			**0.750**	0.030*	**0.531**	0.031*	**0.865**	0.030*
B	0.458	0.025*	0.010	0.430			**0.844**	0.028*	**0.938**	0.028*
C	0.688	0.029*	0.010	0.403	0.021	0.371			**0.604**	0.030*
D	0.677	0.029*	0.354	0.030*	0.188	0.145	0.188	0.181		

Analysis of similarity was calculated between all treatments based on OTUs tables relative (bold font) and absolute abundance (regular font) Bray–Curtis distance matrices. Each pairwise comparison of two groups was performed using 999 permutations. *R* values > 0.75 are generally interpreted as clearly separated, *R* > 0.5 as separated and *R* < 0.25 as groups hardly separated (Krych et al., 2013). CK: 0 ml of PT13; A: 1 ml; B: 5 ml; C: 10 ml; D: 20 ml. *p < 0.05.

### Iconic species under the predation of myxobacteria

**Figure 5:**
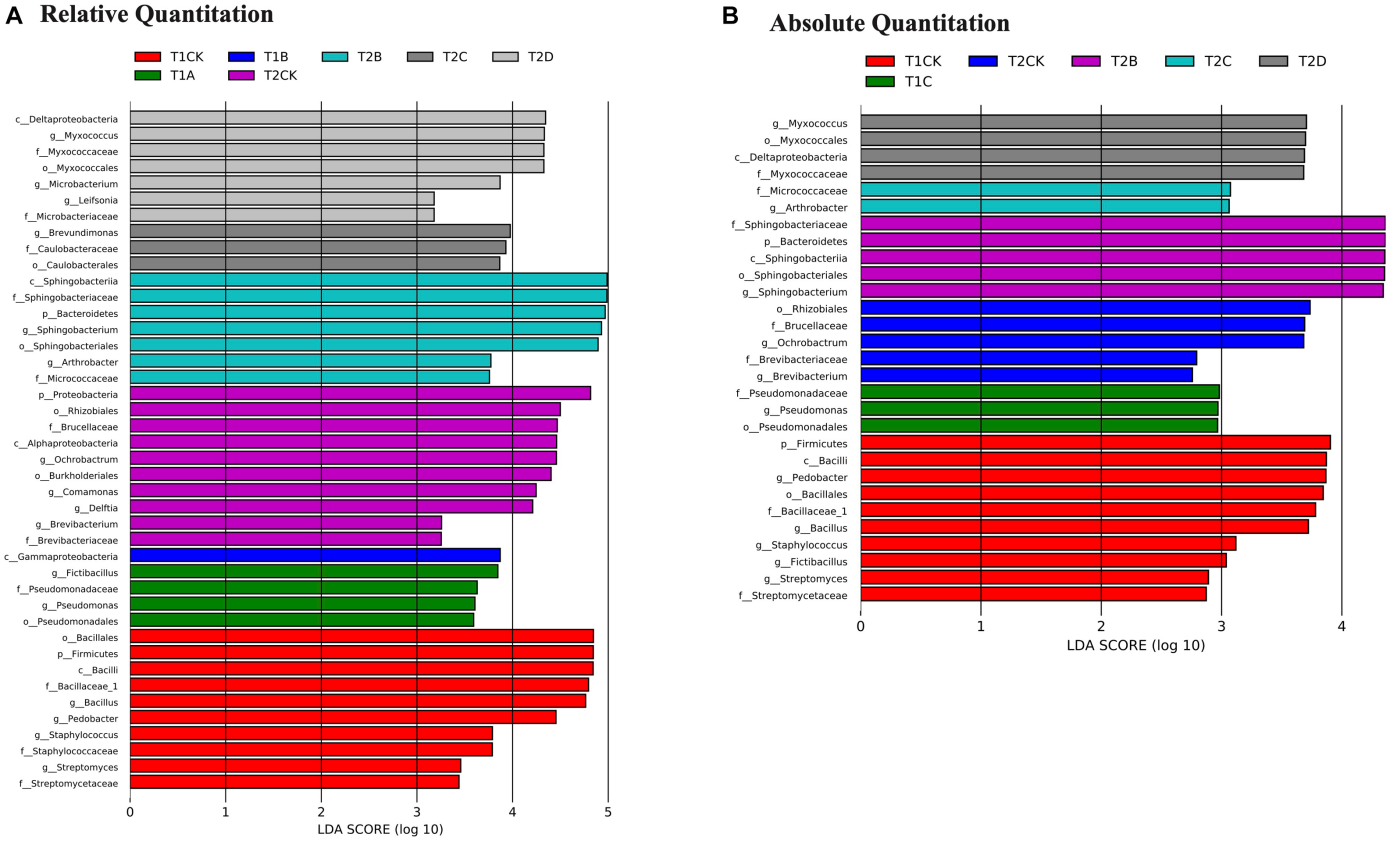
LEfSe plots showing bacterial abundance **(A)** and copies **(B)** enriched in the different treatments. Histograms of different colors stand for taxa which were abundant in the corresponding treatment sample (*p* < 0.05). The different sample numbers indicate the incubation time and the amount of PT13 added. T1: 12 h; T2: 24 h; CK: 0 ml of PT13; A: 1 ml; B: 5 ml; C: 10 ml; D: 20 ml.

LEfSe analysis (Figure 5) was used to distinguish iconic species with significant differences in abundance or copies between the above treatments. The relative abundance results (Figure 5A) showed that a total of 42 bacterial taxa were detected, including four genera (*Bacillus, Pedobacter, Staphylococcus* and *Streptomyces*) in T1CK; four genera in T2CK (*Ochrobactrum, Comamonas, Delftia* and *Brevibacterium*); two genera (*Fictibacillus* and *Pseudomonas*) in T1A; two genera (*Sphingobacterium* and *Paenarthrobacter*) in T2B; one genus (*Brevundimonas*) in T2C; and three genera (*Myxococcus*, *Microbacterium,* and *Leifsonia*) in T2D. A total of 13 genera were detected as iconic microorganisms (Figure 5A, *p* < 0.05). The absolute abundance results (Figure 5B) showed 29 bacterial taxa, including five genera (*Pedobacter*, *Bacillus*, *Staphylococcus*, *Fictibacillus,* and *Streptomyces*) in the T1CK; two genera (*Ochrobactrum* and *Brevibacterium*) in the T2CK; one genus (*Sphingobacterium*) in the T2B; two genera (*Pseudomonas* and *Paenarthrobacter*) in the T1C and T2C treatments; and one genus (*Myxococcus*) in the T2D. A total of 11 iconic genera were detected between these treatments (Figure 5B, *p* < 0.05).

Combining the differential iconic species data for relative and absolute abundance, 13 and 11 iconic genera were disturbed by PT13, respectively. *Myxococcus* sp. PT13 significantly reduced the relative abundance of eight genera and increased the absolute abundance of seven genera (*p* < 0.05) but also increased the relative or absolute abundance of some species, such as *Sphingobacterium*, *Paenarthrobacter*, *Microbacterium*, and *Leifsonia* (Figure 5A).

## Discussion

Myxobacteria are the most common predatory bacteria in agricultural soils, and their good motility and sociological behavior have attracted attention from researchers (Thiery and Kaimer, 2020; Wang et al., 2020a). *Myxococcus* sp. PT13, a wild myxobacteria strain isolated from yellow-brown soils, was chosen for its good bacterial lysing ability and motility. The lysis spectrum of PT13 was determined by measuring the predation diameter of 62 typical soil strains, including *Aeromonas*, *Bacillus, Brevibacterium*, *Fictibacillus*, *Glutamicibacter*, *Herbaspirillum,* and *Leifsonia.* Gram positivity or negativity does not directly affect its lysis effect, and the above conclusions are similar to the results of Dai et al. (2020). In addition, our study measured multiple strains of the same genus, such as the diameter of *Bacillus* sp. TF was preyed upon up to 26.7 mm, whereas *Bacillus* sp. TA and TN were largely not predated. This suggests that strain differences directly influence predation diameter (efficiency). Similar results were shown for *Microbacterium*, *Paenarthrobacter*, and *Pseudomonas* (Figure 1).

*Bacillus licheniformis* TN can significantly resist the predation of PT13 (Figure 1). The findings also afford additional evidence that *Bacillus licheniformis* escapes from *M. xanthus* predation by deactivating myxovirescin A through enzymatic glucosylation (Wang et al., 2019). There is also evidence that bacillaene inhibits *M. xanthus* predation and sporulation protects *Bacillus subtilis* from predation by *M. xanthus* (Müller et al., 2014). In addition, *Bacillus subtilis* can produce an extracellular matrix and biofilm to defend against *M. xanthus* (Susanne et al., 2015). Akbar et al. observed some surviving *Pseudomonas* phenotypes able to elude *M. xanthus* predation. Increased pyoverdine production, mucoid conversion, and antibiotic resistance observed from survivor *Pseudomonas putida* associated with avoidance of the *M. xanthus* predation (Akbar and Stevens, 2021). *Sinorhizobium meliloti* utilizes secreted Galactoglucan protects cells from *M. xanthus* (Pérez et al., 2014). In addition, there are many factors related to the predator avoidance of prey bacteria, including quorum sensing (Sun et al., 2013; Shukria et al., 2021), increasing the amount of mucus and reducing the movement speed of myxobacteria (Nair et al., 2019), toxin production functional genomics (Weitere et al., 2010; Akbar and Stevens, 2021), type III and type VI secretion systems (Coulthurst, 2019; Le et al., 2021) and antibiotic resistance-associated efflux pumps (Ana et al., 2017). We speculate that these factors may be related to the characteristics of the strain itself, resulting in the difference in the predation efficiency of PT13 on the prey strains of the same genus.

However, the present results do not support the idea that myxobacteria have a greater preference for predation on gram-negative prey bacteria (Morgan et al., 2010; Mendes-Soares and Velicer, 2013; Livingstone et al., 2017; Petters et al., 2021). However, the 20 strains of *Streptomyces* spp. that were gram-positive significantly restricted the motility and lysis of PT13 (Figure 1). This may be related to the fact that both are important medicinal microorganisms. Myxobacteria are another important drug-derived microbial group after *Streptomyces* (actinomycetes), which can produce abundant secondary metabolites (Iizuka et al., 2013). There is also evidence that *Streptomyces coelicolor* M45 resists predation by *M. xanthus* DK1622 through aerial mycelia and antimicrobial substances (Pérez et al., 2011, 2016). Lee et al. (2020) found that iron competition triggered antibiotic biosynthesis in *Streptomyces coelicolor* during coculture with *M. xanthus*. In the soil, both *Myxococcus* and *Streptomyces* coexist and there is evidence of horizontal gene (*celA* gene) transfer between *Streptomyces* and *Myxococcus* ancestors (Quillet et al., 1995; Pérez et al., 2011). In conclusion, *Myxococcus* PT13 has a significant lytic effect on typical agricultural soil bacteria, but its preference is linked to the strain itself, and *Streptomyces* can effectively inhibit the lysis of PT13.

A microcosmic system composed of 16 indigenous bacteria was constructed, and myxobacteria PT13 could prey on these bacteria in the microcosmic system and eventually colonize. This result showed that the interaction of multiple prey bacteria cannot completely resist predation by PT13. However, there was a preference for lysing these prey bacteria by PT13, e.g., the relative and absolute abundances (copy numbers) of *Bacillus*, *Pedobacter*, *Staphylococcus*, *Streptomyces* and *Fictibacillus* were significantly reduced for PT13 addition. In particular, *Streptomyces* was significantly antagonistic to lysis by PT13 under one-to-one predation but was significantly lysed under this microcosmic system. This may be related to the culture environment (solid plates, liquid shake flasks) in which they were incubated or to the interaction of several microorganisms.

Myxobacteria are generally considered to be the apex predators of these groups. The nutrients released by the prey of myxobacteria not only maintain the growth of myxobacteria but also increase the absolute copy number of other bacteria, which is manifested in *Sphingobacterium*, *Pseudomonas* and *Paenarthrobacter*. However, the increase in relative abundance in the study of Dai et al. was only manifested in the genus *Burkholderia*. This paper further elaborates the above results from the perspective of absolute copy number and reconfirmed that myxobacteria does not significantly reduce the bacterial community richness indices (ACE and Chao1).

In complex microcosmic systems, bacterial community assembly processes often have multiple mechanisms, such as heterogeneous selection (HeS), homogeneous selection (HoS), dispersal limitation (DL), homogenizing dispersal (HD), and drift (DR) (Ning et al., 2020). In addition, some scholars suggest that priority effects by the initially inoculated community reduce the establishment success of taxa from the later arriving community (Svoboda et al., 2018). The predation of PT13 is likely involved in the community assembly process of bacteria but not necessarily, population drift and other mechanisms have potential effects on this system.

Myxobacteria are generally considered to be microbial predators with broad-spectrum lysis capabilities in soil. Notably, there are many factors in the soil environment that limit their habitat. Examples include excessive use of nitrogen fertilizers, incompatibility between individuals of different myxobacteria (potential inhibition), and inhibition of other predatory microorganisms (*Streptomyces* spp. etc.) (Wang et al., 2020b). We noticed a study on *Bdellovibrio* (obligate predatory bacteria), where they constructed prey landscapes including periplasmic or epibiotic predators including two types of decoy under a large range of initial decoy:prey ratio, and mixed cultures containing multiple predators and prey (Sathyamoorthy et al., 2021). They believe that in complex prey landscapes, such as multiple predator and prey cultures, less preferred prey appears to act as decoy (Sathyamoorthy et al., 2021). This study partly explains the coexistence of PT13 with some microorganisms in the microcosmic system. This paper adopted a microcosmic system to confirm the predation preference of myxobacteria under complex microbial interactions. This predation preference preserves other potential prey bacteria and is an important factor in the coexistence of some prey bacteria and myxobacteria.

## Conclusion

In this study, we explored the lysis spectrum of *Myxococcus* sp. PT13 on different typical soil bacteria and clarified the disturbance of the bacterial community structure in the microcosmic system by myxobacteria predation. PT13 has a preference for predation of soil bacteria and a significant lysis effect on the genera *Bacillus, Brevibacterium*, *Herbaspirillum,* and *Leifsonia* but poor lysis effect on 20 *Streptomyces* spp. In the microcosmic system constructed by 16 indigenous prey bacteria, the predation of PT13 was likely the main factor driving the bacterial communities. The added volume of PT13 was also an important factor affecting the bacterial community composition. However, there are many factors affecting the predation of myxobacteria in the actual soil environment, and this paper adopts a simplified microcosmic system to focus on the interaction between microorganisms, thereby ignoring the influence of other factors, which is somewhat insufficient. However, this study further enriches the knowledge and understanding of predatory myxobacteria in soil habitats and lays a theoretical foundation for the study of the regulation of soil microecology by myxobacteria.

## Data availability statement

The datasets presented in this study can be found in online repositories. The names of the repository/repositories and accession number(s) can be found in the article/Supplementary material.

## Author contributions

YY, HT, and WM performed all the experiments and coordinated the data analyses. WW prepared the manuscript, experimental design, and formal analysis. WW and YY contributed to the preparation of the manuscript and data analyses. WW and XC conceived the research. XC revised the manuscript. WW and NW supervised the entire study. All authors contributed to the article and approved the submitted version.

## Funding

This work was financially supported by Anhui Provincial Natural Science Foundation, China (2108085QC89), the Natural Science Research Project of Anhui Educational Committee, China (2022AH050870), and the Anhui Postdoctoral Science Foundation, China (2020B410).

## Conflict of interest

The authors declare that the research was conducted in the absence of any commercial or financial relationships that could be construed as a potential conflict of interest.

## Publisher’s note

All claims expressed in this article are solely those of the authors and do not necessarily represent those of their affiliated organizations, or those of the publisher, the editors and the reviewers. Any product that may be evaluated in this article, or claim that may be made by its manufacturer, is not guaranteed or endorsed by the publisher.

## Footnotes

^1^ https://www.megasoftware.net/home

## Supplementary material

SUPPLEMENTARY FIGURE S1 Effect of myxobacteria addition on microcosmic bacterial community diversity indices.

SUPPLEMENTARY FIGURE S2 Absorbance of microcosmic systems with different incubation times.

SUPPLEMENTARY TABLE S1 16S rRNA sequence information of 62 soil bacteria.

## References

[ref1] Akbar S. Stevens D. C. (2021). Functional genomics study of *Pseudomonas putida* to determine traits associated with avoidance of a myxobacterial predator. Sci. Rep. 11:16445. doi: 10.1038/s41598-021-96046-8, PMID: 34385565 PMC8360965

[ref2] Ana V. Amritha R. Alan M. S. Andrey V. K. (2017). CmeABC multidrug efflux pump contributes to antibiotic resistance and promotes *Campylobacter jejuni* survival and multiplication in *Acanthamoeba polyphaga*. Appl. Environ. Microbiol. 83:e01600-17. doi: 10.1128/AEM.01600-17 28916560 PMC5666138

[ref3] Anderson M. J. Walsh D. C. I. (2013). PERMANOVA, ANOSIM, and the mantel test in the face of heterogeneous dispersions: what null hypothesis are you testing? Ecol. Monogr. 83, 557–574. doi: 10.1890/12-2010.1

[ref4] Arend K. I. Schmidt J. J. Bentler T. Lüchtefeld C. Eggerichs D. Hexamer H. M.. (2021). *Myxococcus xanthus* predation of gram-positive or gram-negative bacteria is mediated by different bacteriolytic mechanisms. Appl. Environ. Microbiol. 87, e02382–e02320. doi: 10.1128/AEM.02382-20, PMID: 33310723 PMC8090889

[ref5] Berleman J. E. Kirby J. R. (2009). Deciphering the hunting strategy of a bacterial wolfpack. FEMS Microbiol. Rev. 33, 942–957. doi: 10.1111/j.1574-6976.2009.00185.x, PMID: 19519767 PMC2774760

[ref6] Bhat M. A. Mishra A. K. Bhat M. A. Banday M. I. Bashir O. Rather I. A.. (2021). Myxobacteria as a source of new bioactive compounds: a perspective study. Pharmaceutics 13:1265. doi: 10.3390/pharmaceutics13081265 34452226 PMC8401837

[ref7] Bull C. T. Shetty K. G. Subbarao K. V. (2002). Interactions between Myxobacteria, plant pathogenic fungi, and biocontrol agents. Plant Dis. 86, 889–896. doi: 10.1094/PDIS.2002.86.8.889, PMID: 30818644

[ref8] Coulthurst S. (2019). The type VI secretion system: a versatile bacterial weapon. Microbiology 165, 503–515. doi: 10.1099/mic.0.000789, PMID: 30893029

[ref9] Dai W. Jiu M. Wang W. Cui Z. Wang H. (2020). Effects of myxobacteria predation on microbial community structure of artificial microcosm. Acta Microbiol Sin. 60, 452–463.

[ref10] Iizuka T. Jojima Y. Hayakawa A. Fujii T. Yamanaka S. Fudou R. (2013). *Pseudenhygromyxa salsuginis* gen. Nov., sp. nov., a myxobacterium isolated from an estuarine marsh. Int. J. Syst. Evol. Microbiol. 63, 1360–1369. doi: 10.1099/ijs.0.040501-0, PMID: 22821734

[ref11] Krych L. Hansen C. H. Hansen A. K. Van Den Berg F. W. Nielsen D. S. (2013). Quantitatively different, yet qualitatively alike: a meta-analysis of the mouse core gut microbiome with a view towards the human gut microbiome. PLoS One 8:e62578. doi: 10.1371/journal.pone.0062578, PMID: 23658749 PMC3641060

[ref12] Le N. H. Pinedo V. Lopez J. Cava F. Feldman M. F. (2021). Killing of gram-negative and gram-positive bacteria by a bifunctional cell wall-targeting T6SS effector. Natl. Acad. Sci. 118:e2106555118. doi: 10.1073/pnas.2106555118 PMC8501793 34588306

[ref13] Lee N. Kim W. Chung J. Lee Y. Cho S. Jang K. S.. (2020). Iron competition triggers antibiotic biosynthesis in *Streptomyces coelicolor* during coculture with *Myxococcus xanthus*. ISME J. 14, 1111–1124. doi: 10.1038/s41396-020-0594-6, PMID: 31992858 PMC7174319

[ref14] Letunic I. Bork P. (2016). Interactive tree of life (iTOL) v3: an online tool for the display and annotation of phylogenetic and other trees. Nucleic Acids Res. 44, W242–W245. doi: 10.1093/nar/gkw290, PMID: 27095192 PMC4987883

[ref15] Li Z. Wang T. Luo X. Li X. Xia C. Zhao Y.. (2018). Biocontrol potential of *Myxococcus* sp. strain BS against bacterial soft rot of calla lily caused by *Pectobacterium carotovorum*. Biol. Control 126, 36–44. doi: 10.1016/j.biocontrol.2018.07.004

[ref16] Li Z. Ye X. Yang F. Huang Y. Fan J. Wang H.. (2021). The predation biology of myxobacteria and its application in agricultural field. J. Nanjing Agric. Univ. 44, 208–216. doi: 10.7685/jnau.202010034

[ref17] Li Y. Zhou X. Zhang X. Xu Z. Dong H. Yu G.. (2022). A myxobacterial GH19 lysozyme with bacteriolytic activity on both gram-positive and negative phytopathogens. AMB Express 12:54. doi: 10.1186/s13568-022-01393-y, PMID: 35551524 PMC9098779

[ref18] Liu Y. H. Liu B. B. Li W. J. (2021). Isolation and preservation of microorganisms in salty Lake. Microbiome Protocols eBook. Bio-101:e2003811. doi: 10.21769/BioProtoc.2003811

[ref19] Liu Y. Yao Q. Zhu H. (2019). Meta-16S rRNA gene phylogenetic reconstruction reveals the astonishing diversity of cosmopolitan Myxobacteria. Microorganisms 7:551. doi: 10.3390/microorganisms7110551 31717918 PMC6920832

[ref20] Livingstone P. G. Morphew R. M. Whitworth D. E. (2017). Myxobacteria are able to prey broadly upon clinically-relevant pathogens, exhibiting a prey range which cannot be explained by phylogeny. Front. Microbiol. 8:1593. doi: 10.3389/fmicb.2017.01593, PMID: 28878752 PMC5572228

[ref21] Lloyd D. G. Whitworth D. E. (2017). The myxobacterium *Myxococcus xanthus* can sense and respond to the quorum signals secreted by potential prey organisms. Front. Microbiol. 8:439. doi: 10.3389/fmicb.2017.00439 28352265 PMC5348527

[ref22] Mendes-Soares H. Velicer G. J. (2013). Decomposing predation: testing for parameters that correlate with predatory performance by a social bacterium. Microb. Ecol. 65, 415–423. doi: 10.1007/s00248-012-0135-6, PMID: 23184156 PMC3563865

[ref23] Morgan A. D. MacLean R. C. Hillesland K. L. Velicer G. J. (2010). Comparative analysis of Myxococcus predation on soil Bacteria. Appl. Environ. Microbiol. 76, 6920–6927. doi: 10.1128/AEM.00414-10, PMID: 20802074 PMC2953020

[ref24] Müller S. Strack S. N. Hoefler B. C. Straight P. D. Kearns D. B. Kirby J. R. (2014). Bacillaene and sporulation protect *Bacillus subtilis* from predation by *Myxococcus xanthus*. Appl. Environ. Microbiol. 80, 5603–5610. doi: 10.1128/aem.01621-14, PMID: 25002419 PMC4178607

[ref25] Nair R. R. Vasse M. Wielgoss S. Sun L. Yu Y.-T. N. Velicer G. J. (2019). Bacterial predator-prey coevolution accelerates genome evolution and selects on virulence-associated prey defences. Nat. Commun. 10:4301. doi: 10.1038/s41467-019-12140-6, PMID: 31541093 PMC6754418

[ref26] Ning D. Yuan M. Wu L. Zhang Y. Guo X. Zhou X.. (2020). A quantitative framework reveals ecological drivers of grassland microbial community assembly in response to warming. Nat. Commun. 11:4717. doi: 10.1038/s41467-020-18560-z, PMID: 32948774 PMC7501310

[ref27] Pérez J. Jiménez-Zurdo J. I. Martínez-Abarca F. Millán V. Shimkets L. J. Muñoz-Dorado J. (2014). Rhizobial galactoglucan determines the predatory pattern of *Myxococcus xanthus* and protects *Sinorhizobium meliloti* from predation. Environ. Microbiol. 16, 2341–2350. doi: 10.1111/1462-2920.12477, PMID: 24707988 PMC4079745

[ref28] Pérez J. Moraleda-Muñoz A. Marcos-Torres F. J. Muñoz-Dorado J. (2016). Bacterial predation: 75 years and counting! Environ. Microbiol. 18, 766–779. doi: 10.1111/1462-2920.13171, PMID: 26663201

[ref29] Pérez J. Muñoz-Dorado J. Braña A. F. Shimkets L. J. Sevillano L. Santamaría R. I. (2011). *Myxococcus xanthus* induces actinorhodin overproduction and aerial mycelium formation by *Streptomyces coelicolor*. Microb. Biotechnol. 4, 175–183. doi: 10.1111/j.1751-7915.2010.00208.x, PMID: 21342463 PMC3818858

[ref30] Petters S. Groß V. Söllinger A. Pichler M. Reinhard A. Bengtsson M. M.. (2021). The soil microbial food web revisited: predatory myxobacteria as keystone taxa? ISME J. 15, 2665–2675. doi: 10.1038/s41396-021-00958-2, PMID: 33746204 PMC8397742

[ref31] Phillips K. E. Akbar S. Stevens D. C. (2022). Concepts and conjectures concerning predatory performance of myxobacteria. Front. Microbiol. 13:1031346. doi: 10.3389/fmicb.2022.1031346, PMID: 36246230 PMC9556981

[ref32] Quillet L. Barray S. Labedan B. Petit F. Guespin-Michel J. (1995). The gene encoding the β-1,4-endoglucanase (CelA) from *Myxococcus xanthus*: evidence for independent acquisition by horizontal transfer of binding and catalytic domains from actinomycetes. Gene 158, 23–29. doi: 10.1016/0378-1119(95)00091-J, PMID: 7789807

[ref33] Sathyamoorthy R. Huppert A. Kadouri D. E. Jurkevitch E. (2021). Effects of the prey landscape on the fitness of the bacterial predators Bdellovibrio and like organisms. FEMS Microbiol. Ecol. 97:fiab047. doi: 10.1093/femsec/fiab047, PMID: 33739375

[ref34] Segata N. Izard J. Waldron L. Gevers D. Miropolsky L. Garrett W. S.. (2011). Metagenomic biomarker discovery and explanation. Genome Biol. 12:R60. doi: 10.1186/gb-2011-12-6-r60, PMID: 21702898 PMC3218848

[ref35] Shukria A. Sandeep K. M. Joshua S. S. Stevens D. C. (2021). Differential response to prey quorum signals indicates predatory range of myxobacteria. bioRxiv:2021.2006.2004.447097. doi: 10.1101/2021.06.04.447097

[ref36] Stoddard S. F. Smith B. J. Hein R. Roller B. R. Schmidt T. M. (2015). rrnDB: improved tools for interpreting rRNA gene abundance in bacteria and archaea and a new foundation for future development. Nucleic Acids Res. 43, D593–D598. doi: 10.1093/nar/gku1201, PMID: 25414355 PMC4383981

[ref37] Sun S. Kjelleberg S. McDougald D. (2013). Relative contributions of Vibrio polysaccharide and quorum sensing to the resistance of *Vibrio cholerae* to predation by heterotrophic Protists. PLoS One 8:e56338. doi: 10.1371/journal.pone.0056338, PMID: 23441178 PMC3575383

[ref38] Susanne M. Sarah N. S. Sarah E. R. Daniel B. K. John R. K. (2015). Predation by *Myxococcus xanthus* induces *Bacillus subtilis* to form spore-filled megastructures. Appl. Environ. Microbiol. 81, 203–10. doi: 10.1128/AEM.02448-14 25326308 PMC4272737

[ref39] Svoboda P. Lindström E. S. Ahmed Osman O. Langenheder S. (2018). Dispersal timing determines the importance of priority effects in bacterial communities. ISME J. 12, 644–646. doi: 10.1038/ismej.2017.180, PMID: 29053147 PMC5776460

[ref40] Thiery S. Kaimer C. (2020). The predation strategy of *Myxococcus xanthus*. Front. Microbiol. 11:2. doi: 10.3389/fmicb.2020.00002, PMID: 32010119 PMC6971385

[ref41] Wang C. Liu X. Zhang P. Wang Y. Li Z. Li X.. (2019). *Bacillus licheniformis* escapes from *Myxococcus xanthus* predation by deactivating myxovirescin a through enzymatic glucosylation. Environ. Microbiol. 21, 4755–4772. doi: 10.1111/1462-2920.14817, PMID: 31600864

[ref42] Wang W. Luo X. Ye X. Chen Y. Wang H. Wang L.. (2020a). Predatory Myxococcales are widely distributed in and closely correlated with the bacterial community structure of agricultural land. Appl. Soil Ecol. 146:103365. doi: 10.1016/j.apsoil.2019.103365

[ref43] Wang W. Wang N. Dang K. Dai W. Guan L. Wang B.. (2020b). Long-term nitrogen application decreases the abundance and copy number of predatory myxobacteria and alters the myxobacterial community structure in the soil. Sci. Total Environ. 708:135114. doi: 10.1016/j.scitotenv.2019.135114, PMID: 31812411

[ref44] Wang J. Wang J. Wu S. Zhang Z. Li Y. (2021). Global geographic diversity and distribution of the Myxobacteria. Microbiol. Spectr. 9, e0001221–e0000021. doi: 10.1128/Spectrum.00012-21, PMID: 34259548 PMC8552515

[ref45] Weitere M. Bergfeld T. Rice S. A. Matz C. Kjelleberg S. (2010). Grazing resistance of *Pseudomonas aeruginosa* biofilms depends on type of protective mechanism, developmental stage and protozoan feeding mode. Environ. Microbiol. 7, 1593–1601. doi: 10.1111/j.1462-2920.2005.00851.x 16156732

[ref46] Whitworth D. E. Zwarycz A. (2020). A genomic survey of signalling in the Myxococcaceae. Microorganisms 8:1739. doi: 10.3390/microorganisms8111739, PMID: 33171896 PMC7694542

[ref47] Ye X. Li Z. Luo X. Wang W. Li Y. Li R.. (2020). A predatory myxobacterium controls cucumber fusarium wilt by regulating the soil microbial community. Microbiome 8:49. doi: 10.1186/s40168-020-00824-x, PMID: 32252828 PMC7137222

[ref48] Yi S. Zhou Y. Zhang X. Yao Q. Li H. Zhu H. (2021). Effects of different methods on the formation of fruiting bodies and isolation of myxobacteria. Acta Microbiol. Sin. 61, 923–934.

[ref49] Zhou Y. Yi S. Zhang X. Yao Q. Honghui Z. (2020). Isolation of soil myxobacteria based on bacterial co⁃occurrence network. Biotic Resour. 42, 531–539. doi: 10.14188/j.ajsh.2020.05.007

