# Supplementary material for: Lysis profile and preference of Myxococcus sp. PT13 for typical soil bacteria
Source: Front Microbiol. 2023 Jun 12;14:1211756. doi: 10.3389/fmicb.2023.1211756 (PMC10291197; doi:10.3389/fmicb.2023.1211756)
Supplement: SUPPLEMENTARY FIGURE S1 — Effect of myxobacteria addition on microcosmic bacterial community diversity indices. [file Data_Sheet_1.DOCX]

**Title Page**

**Type of contribution:** regular paper

**Date of preparation:** 10 February 2023

**Number of text pages:** 24, **number of tables:** 3, **number of figures:** 5

**Number of Supplementary tables:** 1, **Number of Supplementary figures:** 2

**Title:** Lysis profile and preference of *Myxococcus* sp. PT13 for typical soil bacteria

**Author names:** Yi Yang^1^, Hong Tao^1^,Wenwen Ma^1^, Nana Wang^1^, Xiaolin Chen^1^, and Wenhui Wang^1*^

**Affiliation:**

^1^School of Life Sciences, Anhui Agricultural University, Hefei, Anhui 230036, China

***Corresponding author**: Wenhui Wang

**Corresponding address:** School of Life Sciences, Anhui Agricultural University, Hefei, Anhui 230036, China

**Tel.:** +86055165786319, **Fax:** +86055165786319, **E-mail:** [wangwenhui@ahau.edu.cn](mailto:wangwenhui@ahau.edu.cn)

**Supplementary information I**

**Supplementary Figures and Tables**

**Table S1. 16S rRNA sequence information of 62 soil bacteria.**

**Please find Table S1 in the supplement Excel file.**

**Figure S1.**

**Figure S1. Effect of myxobacteria addition on microcosmic bacterial community diversity indices.**

The different sample numbers indicate the incubation time and the volume of PT13 added. T1: 12 hours; T2: 24 hours; CK: 0 mL of PT13; A: 1 mL; B: 5 mL; C: 10 mL; D: 20 mL.

**Figure S2.**


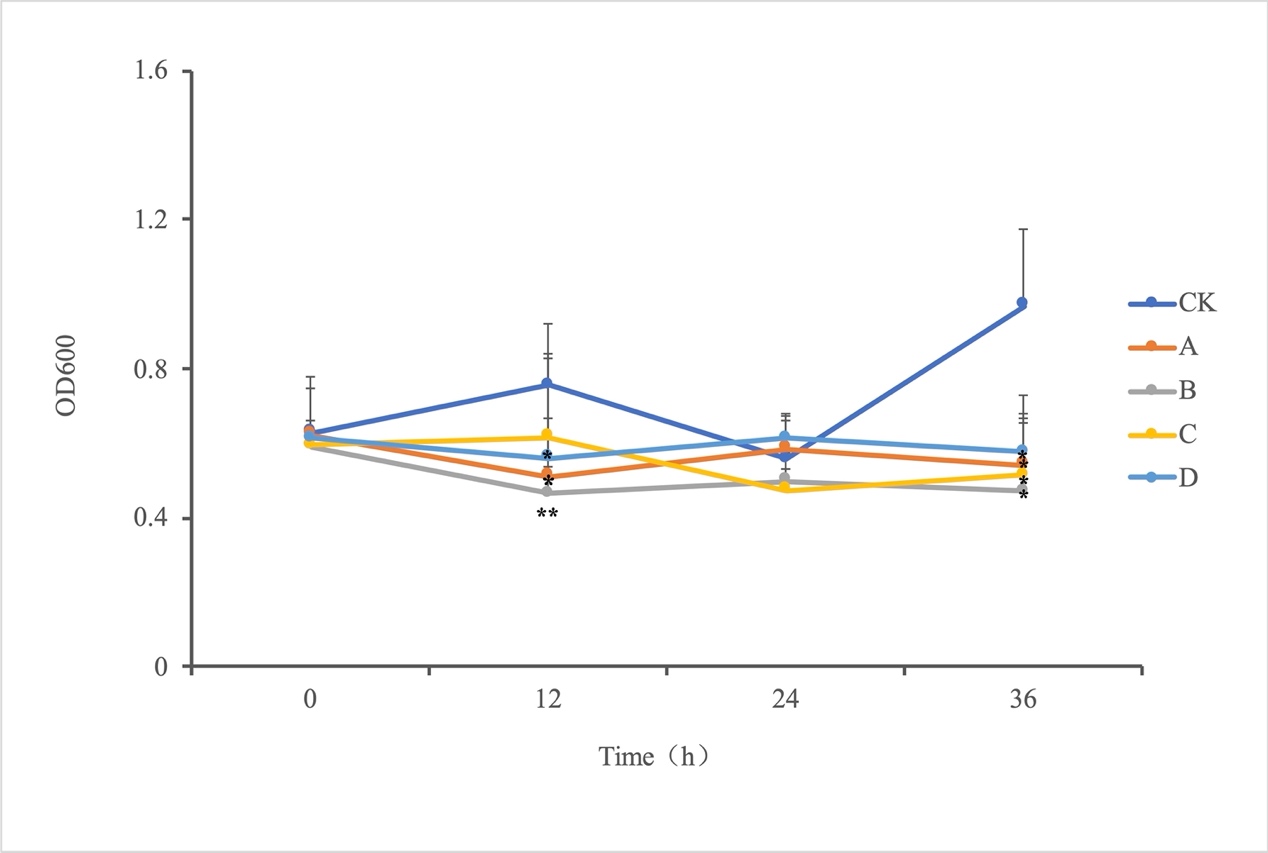


**Figure S2. Absorbance of microcosmic systems with different incubation times.**

The different sample numbers indicate the incubation time and the volume of PT13 added. CK: 0 mL of PT13; A: 1 mL; B: 5 mL; C: 10 mL; D: 20 mL (average proportion, n = 3). Note: ANOVA, ref.group = "CK", **P* < 0.05, ***P* < 0.01.
